# Mating behaviour, mate choice and female resistance in the bean flower thrips (*Megalurothrips sjostedti*)

**DOI:** 10.1038/s41598-021-93891-5

**Published:** 2021-07-15

**Authors:** Adeyemi O. Akinyemi, Sevgan Subramanian, David K. Mfuti, Tom W. Pope, Amanuel Tamiru, William D. J. Kirk

**Affiliations:** 1 International Centre of Insect Physiology and Ecology (Icipe), P.O. Box 30772-00100, Nairobi, Kenya; 2 Department of Agronomy, Osun State University, Osogbo, Nigeria; 3 Department of Agriculture and Environment, Centre for Integrated Pest Management, Harper Adams University, Newport, Shropshire, TF10 8NB UK; 4 School of Life Sciences, Keele University, Staffordshire, ST5 5BG UK

**Keywords:** Animal behaviour, Entomology

## Abstract

Many species of thrips (Thysanoptera) in the family Thripidae form mating aggregations, but the adaptive significance of these aggregations and the extent of male and female mate choice is poorly understood. We studied the mating behaviour of the bean flower thrips *Megalurothrips sjostedti* (Trybom) (Thysanoptera: Thripidae), which forms male aggregations and occurs across sub-Saharan Africa. We tested whether males choose mates by female age or mating status. No-choice mating bioassays with one male and one female were used to simulate the way males usually encounter only one female at a time in aggregations in the field. Virgin females violently resisted mating attempts by males, but we found no compelling evidence to establish whether this was indiscriminate or was screening suitable males. Younger males (1–2 days old) did not discriminate females by age (1–2 or 7–10 days old), but older males (7–10 days old) avoided mating with older females. Any male choice by female mating status (virgin or mated) was weak or absent. The mating behaviour of *M. sjostedti* shows broad similarities with that of other thrips species that form aggregations, but also shows some distinct and novel differences, which can help our understanding of the adaptive significance of aggregations.

## Introduction

An understanding of mating behaviour and mate choice is important because it influences reproductive success and thus many aspects of the biology and ecology of a species. In insects, this understanding can be useful for developing new approaches to pest management. Thrips (Thysanoptera) are a cosmopolitan order of very small insects with about 6300 known species^1^. They exhibit many unusual mating behaviours. In sub-social fungus-feeding thrips in the family Phlaeothripidae, these behaviours include male-male fighting^2^, mate guarding^3^ and alternative male mating strategies^4^. In contrast, the family Thripidae has no sub-social species, but it contains most of the pest species that feed on plants, and several form male mating aggregations of tens to hundreds of males, which appear to be mediated by aggregation pheromones^5^. Females typically arrive at the male aggregations, mate and then depart^6^. Such male aggregations have been observed in the genera *Frankliniella*^6,7^, *Megalurothrips*^8,9^, *Parabaliothrips*^10^, *Pezothrips*^11^ and *Thrips*^12,13^. However, there are distinct and unexplained differences in aggregation behaviour between species. For example, male-male fighting has only been observed in aggregations of the western flower thrips *Frankliniella occidentalis* (Pergande)^14^ and the flower thrips *F. intonsa* (Trybom)^15^. The bean flower thrips *Megalurothrips sjostedti* (Trybom) and the closely related Kelly’s citrus thrips *Pezothrips kellyanus* (Bagnall) commonly form aggregations on leaves in the afternoon^8,9,11,16^, with males often stationary, whereas in other species males aggregate on flowers and appear to be more active^8,12^. There are also several common species in which male aggregations have never been reported, such as the melon thrips *T. palmi* Karny, although an aggregation pheromone has been identified^17^. Similarly, in the onion thrips *T. tabaci* Lindeman, although male aggregations have not been reported, male-male fighting has been described^18^. For such species, it is possible that aggregation occurs, but has just not been observed in the field under the right conditions.

A male aggregation for mating is frequently described as a lek. One of the key features of a classical lek is that females have the opportunity to select males^19^, but in thrips aggregations, although there is male fighting in a few species, suggesting some kind of competition for females, there is no evidence of female choice. In *F. occidentalis*, females appear to mate with the first male they encounter and then depart^6,14^. In laboratory bioassays, females mated readily with most males^20^, which also suggests that females are not choosing. Terry and Dyreson^14^ suggested that male *F. occidentalis* could be competing for the best sites on flowers, where females are more likely to land, which would mean that females do not need to choose because preselection by the males themselves has occurred prior to female visits. However, the adaptive significance of male aggregations and male-male fighting in thrips in the family Thripidae remain unclear.

Mate choice within aggregations could be by females or males or both. Mate choice is often assumed to be by females, but there is growing evidence that male mate choice has been underestimated in the past^21,22^, including in lekking species^23^. When female thrips arrive at a male aggregation, there is the potential for them to choose between the many males present, even if there is no evidence that it occurs. However, females typically arrive singly and so for males the choice is usually whether or not to mate rather than a choice between females. In *F. occidentalis*, males avoid mating with recently mated females^20^, so there is some choice, and this may explain why males ignore some females in aggregations^6^. Evolutionary theory suggests that males should not be choosy, because the benefits of choosiness would not outweigh the costs of lost mating opportunities^22,24^ and that male mate choice is less likely to evolve when there is sequential choice^22^.

Mating behaviour and mate choice has been studied for very few of the thrips species that aggregate; study of further species can give more insight into the behaviours involved and establish patterns of similarity and difference. We have studied the mating behaviour of the bean flower thrips *M*. *sjostedti*, which is a major pest of cowpea *Vigna unguiculata* (L.) Walp. and other grain legumes across sub-Saharan Africa^25,26^. The males form aggregations on the leaves, while the females spend most of their time in the flowers^9^. Although two components of a male-produced aggregation pheromone, which attracts both males and females, have been identified and investigated with olfactometer experiments^27^, there have been no close-range studies of the mating behaviour. Our pilot observations indicated that there were some distinct and unexpected differences from the behaviour we observed in *F. occidentalis*^20,28^. Males often avoided mating with older virgin females and virgin females often strongly resisted male mating attempts. This female resistance and associated risk of injury could affect male mate choice; it could also indicate female choice. Our aim was to describe and understand mating behaviour and mate choice in *M. sjostedti*. Based on our pilot observations, we specifically asked the following questions. Do males choose mates by female age? Do males choose mates by female mating status?

## Methods

### Thrips rearing

*Megalurothrips sjostedti* was reared on cleaned pods of French bean (*Phaseolus vulgaris* L.) at the International Centre of Insect Physiology and Ecology (*icipe*) in Nairobi, Kenya. The culture was started and supplemented occasionally with field-collected *M. sjostedti* from cowpea or French bean crops. Adult *M. sjostedti* is black with white wing bases and can be distinguished easily by eye from other common species, such as *F. occidentalis, T. tabaci* or *Hydatothrips adolfifriderici* Karny. The identification was also confirmed morphologically^26^. The culture was maintained in 1-l screw-top plastic jars at 26 ± 2 °C and L12:D12. The jars were ventilated through a hole in the lid, which was closed over a sheet of paper towel to prevent escapes.

Individual virgin adults of known age were obtained by isolating a single mature larva, propupa or pupa in a clear microcentrifuge tube (1.5 ml) to ensure that they remained virgins. The tube was modified to allow ventilation by melting a hole in the top and closing the top over a small piece of paper towel or fine netting. A small section of bean pod was added to each tube for food. The tubes were kept under the same conditions as the main culture and were monitored daily to establish the age (days post emergence) of the adults. Adults that had emerged since the last observation were 0–1 days old. Keeping them for either a further 1 day, 7–9 days or 7–12 days produced adults that were 1–2 days, 7–10 days or 7–13 days old respectively. Male and female adults could be distinguished easily by the shape of the abdomen, which was narrow and parallel-sided in males and wider and more curved in females.

### Laboratory observations and bioassays of mating behaviour

**Figure 1:**
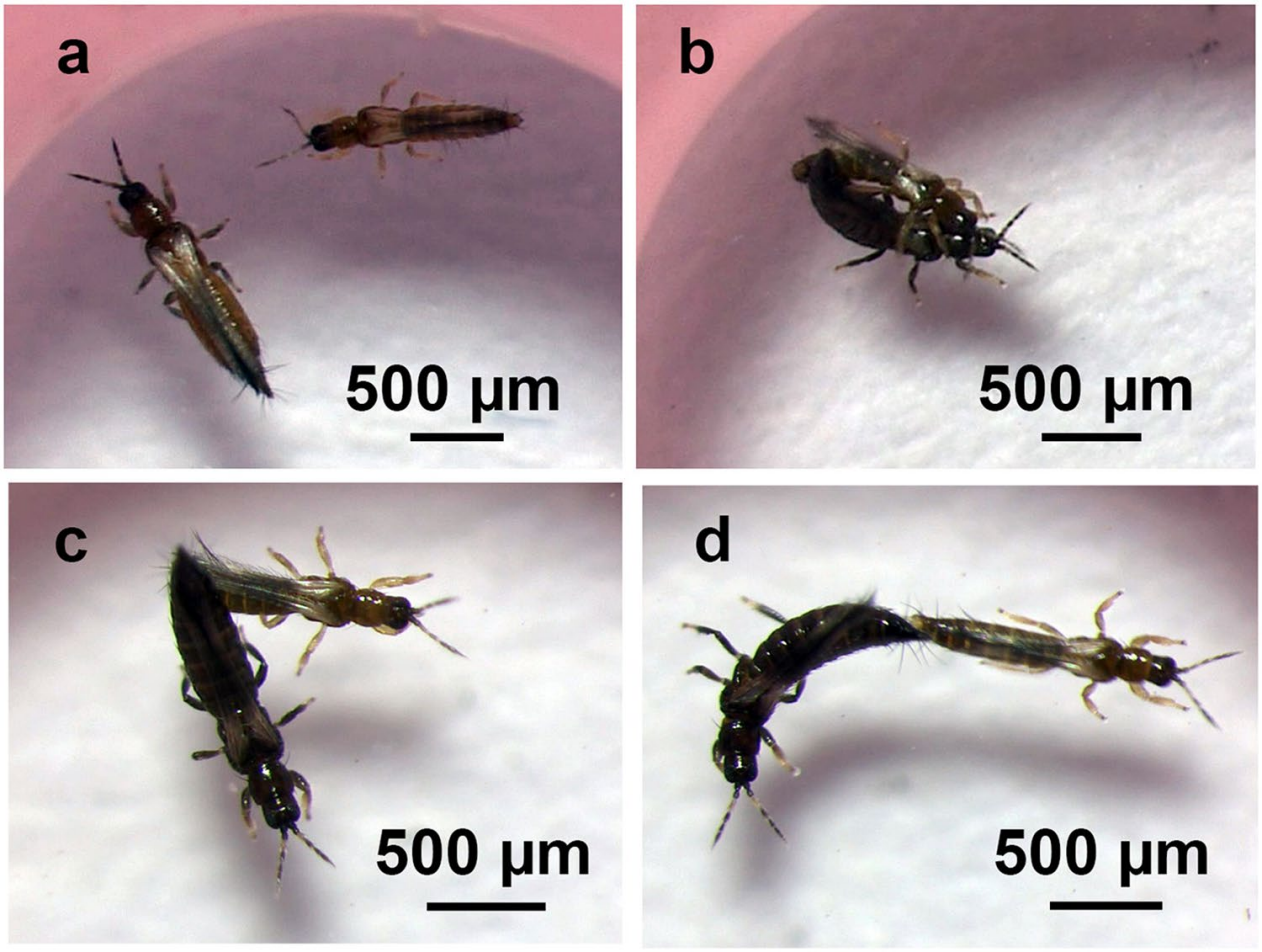
Photographs of the mating behaviour of male and female *Megalurothrips sjostedti*. In each photograph the male is thinner and shorter than the female. Both are virgins. (**a**) Male approaching a female. (**b**) Male on top of a female, antennating and stroking the female with a mid leg while copulating. (**c**) Male and female copulating in the “V”-shape position after antennation and stroking have stopped. (**d**) Male walking away at the end of copulation and pulling at the female abdomen until they separate.

**Figure 2:**
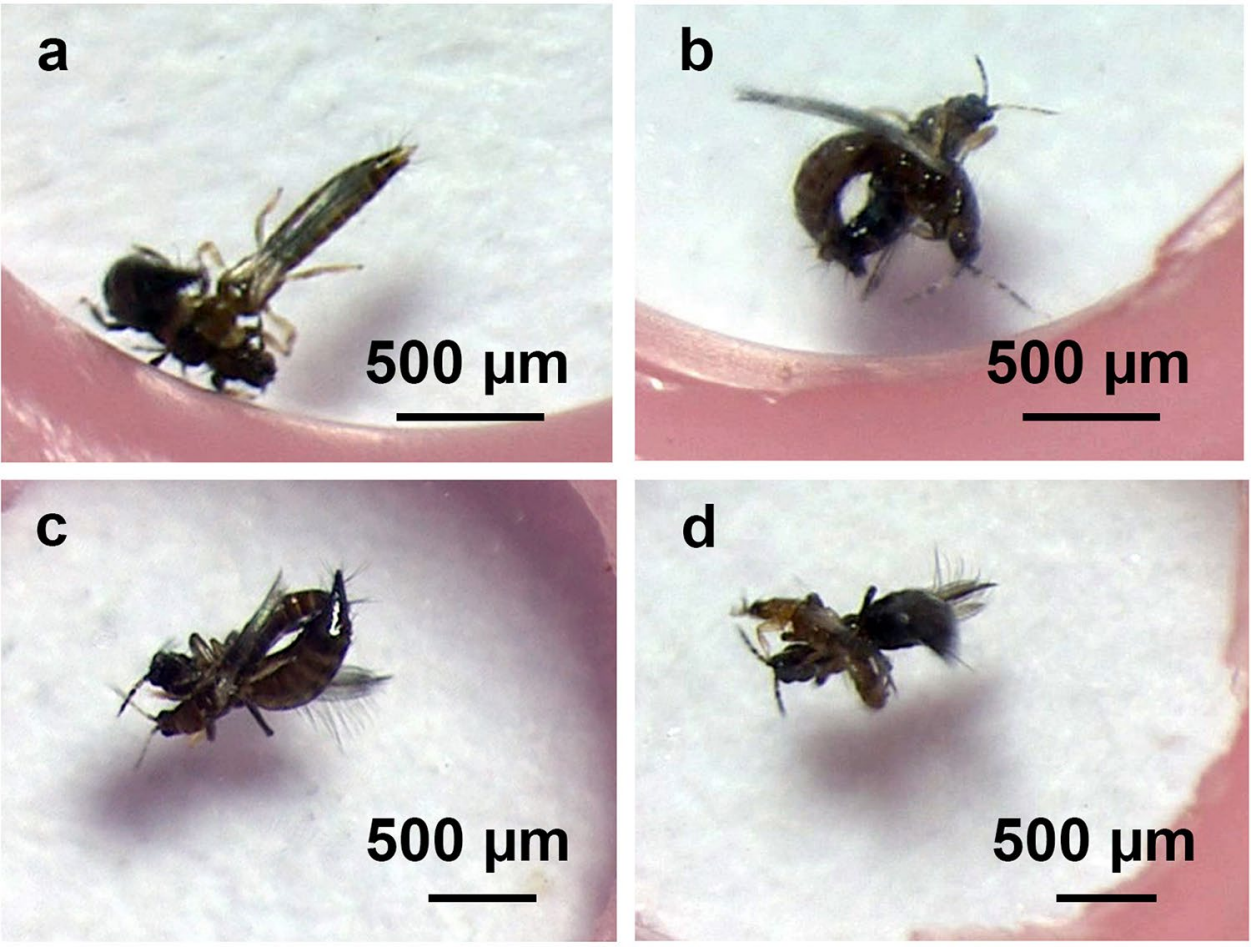
Photographs of female resistance behaviour when a male attempts to copulate with a female *Megalurothrips sjostedti*. In each photograph the male is thinner and shorter than the female. Both are virgins. (**a**) Female flipping its abdomen to resist male. (**b**) Male upside down with the female on top, while the male is attempting to copulate. (**c**,**d**) Female upside down and pressed to the floor by the male attempting to copulate.

In each of the four laboratory experiments, each male and female was observed in a circular arena (diam. 5 mm, height 1.5 mm) cut with a cork borer from a sheet of toughened dental modelling wax (Kemdent, Swindon, UK) and sandwiched between a glass microscope slide base and a glass cover-slip top, as used previously for observations of mating behaviour in *F. occidentalis*^20^. A new arena was used for each pair or, in the experiment with multiple mating events in immediate succession, for each female. Individual thrips were transferred to the arena with a clean, fine brush. Each pair was observed for 10 min or until copulation had finished. The observation period of 10 min was appropriate because males in aggregations only have a short period within which to decide whether to mate and because most pairs that mated did so in the first few minutes. No-choice experiments were used to mirror the situation of sequential choice for males in an aggregation. Choice is sequential because females arrive only occasionally at a male aggregation and the numbers arriving are low compared with the number of males present. Each male therefore encounters and responds to only one female at a time (i.e. sequentially) and it is rare for a male to be in the vicinity of more than one female. Thrips were observed through a Huvitz HSZ-ZB700 or HSZ-TR30 stereomicroscope (Huvitz Corp., South Korea) with LED illumination, either directly by eye or videorecorded with an attached Moticam 1080 full HD camera (Motic, Hong Kong) for later analysis of behaviours and their durations. The photographs for Figs. 1 and 2 and the video for Supplementary Video S1 were taken through a Huvitz HSZ-ZB700 stereomicroscope with a Moticam 1080 full HD camera.

Based on initial observations, we used the same sequence of behaviours as was used previously for *F. occidentalis*^20^, thus allowing inter-specific comparisons. We recorded behaviours as follows: ‘contacted’ (first physical contact, usually occurring in the form of male antennation of the female body); ‘climbed’ (male climbed on the back of the female and aligned its body to face the same direction); ‘bent abdomen’ (male curled its abdomen beneath that of the female while on the back of the female); and ‘copulated’ (contact between the end of the male abdomen and the end of the female abdomen lasting > 5 s). The copulation duration was recorded as the time that the ends of the male and female abdomen were in contact, excluding brief unsuccessful attempts to attach (< 5 s). A few pairs remained copulating for very much longer than the others; the male appeared to be unable to detach because it started to walk away but did not detach as would normally happen. These “stuck” copulations did not reflect the true copulation period, so copulation durations over 900 s were omitted.

Females were considered to be resisting copulation when the male was touching or holding onto the female with its legs and the female was running or moving its body rapidly (e.g. flipping or twisting) in such a way that it was difficult for the male to start copulating. Males often made repeated attempts to copulate, associated with repeated bouts of female resistance. Resistance bouts were timed from the start of resistance until either the female stopped resisting, the male or female separated or copulation commenced, which was considered to occur when the ends of the abdomen were in contact for > 5 s or, if the ends were not in view, when the male abdomen was beneath that of the female for > 5 s. Periods of resistance separated by < 1 s were considered to be part of the same bout. Interactions lasting < 1 s were excluded because they could not be distinguished reliably from collisions. The duration of total female resistance was the sum of the durations of all the bouts of resistance until either copulation started or, if there was no copulation, until the end of the 10 min observation period. Resistance stopped when copulation started, so total resistance was measured over 10 min for those that did not copulate and over less than 10 min for those that did copulate.

This study focused upon the responses of virgin males and females, partly for consistency and partly for easier interpretation of choice. The interests of males and females are more likely to coincide at a virgin female’s first encounter with a potential mate and mate rejection responses by virgin females could be interpreted in terms of mate choice^29^. In all experiments, the treatments (such as male and female ages) were blocked to ensure that treatment was not confounded with day or time of day. This was necessary because thrips activity and responsiveness can change unpredictably between morning and afternoon or between days.

### Field observations of mating behaviour

Observations were made in a plot of cowpea cv ‘Ken-Kunde 1’ at the podding stage with flowers and buds still present (height about 30 cm) grown at the *icipe* Thomas Odhiambo Campus (ITOC) at Mbita, western Kenya (0° 26′ 06.19′′ S, 34° 12′ 53.13′′ E; 1,137 m above sea level). Observations took place during the mornings and afternoons in dry conditions on several days in November 2019 during the short rainy season. The plot was not treated with insecticide. We searched by eye for male aggregations and mating activity on cowpea plants, weeds in the crop, and one blue and two yellow plastic containers (non-baited fruit-fly traps) placed within the crop. Copulation durations were timed with a stopwatch for 14 mating pairs in aggregations on cowpea leaves and plastic surfaces.

### Do males choose mates by female age?

We tested the effect of female age on mating behaviour in two experiments. In the first experiment, the effect of male age was also tested. The species is short-lived as an adult, with half of males dying before 5 days and half of females dying before 13 days^30^, thus our choices of age covered the usual lifespan.

#### Experiment 1

A virgin male, either 1–2 days old or 7–10 days old, was paired with a virgin female, either 1–2 days old or 7–10 days old, in all four age combinations. We recorded the behaviours that occurred and how long they lasted. Each treatment was replicated 19–21 times. The behavioural data from this experiment were also used for the description of mating behaviour.

#### Experiment 2

Experiment 2. We tested the effect of female age on mating behaviour, but prevented any behavioural influence from the female by using dead females. Male thrips are able to copulate with dead females^20,31^. This ensured any choice was determined by the male. A virgin female (1–2 days old or 7–10 days old) was paired with a virgin male (7–10 days old). We used older males because they were shown to be more discriminating in the previous experiment with live females. The virgin females were placed in individual microcentrifuge tubes (1.5 ml) and killed by transferring then to a – 80 °C freezer for 40–45 min and then removed from the freezer and allowed to defrost for at least 10 min. An individual dead female was placed in a 5 mm diameter wax arena, as described above, either standing on its legs or on its side if it would not stay in a standing position, before introducing a male. Males could copulate with females in either position. The behaviours were recorded. Each treatment was replicated 18 times.

### Do males choose mates by female mating status?

#### Experiment 3

We tested whether virgin males would mate with a female that had mated immediately before or 24 h earlier. A virgin male (7–13 days old) was introduced to a virgin female (1–2 days old) (1st introduction). If copulation took place, the male was removed after copulation had finished and a new virgin male (7–13 days old) was introduced to the same female (2nd introduction). If copulation took place, the male was removed after copulation had finished and the twice-mated female was then kept in a microcentrifuge tube (1.5 ml) with a small section of bean pod at 26 ± 2 °C and L12:D12 for 24 h. The female (now 2–3 days old) was paired with a new virgin male (7–13 days old) (3rd introduction) to test if they would copulate. Only females that copulated after an introduction were tested for the next male introduction. The experiment was repeated for 12 females with a total of 33 males.

#### Experiment 4

We further tested the effect of female mating status on male choice by pairing live males with dead females that were either virgin or mated. This prevented any behavioural influence from the female and ensured any choice was determined by the male. Individually isolated virgin females (1–2 days old) from the same cohort were placed individually in an arena, either with a virgin male or alone. Once copulation was completed, the male was removed. Females that copulated and females that were kept alone were killed by immediately transferring the arenas, sealed in aluminium foil, to a − 80 °C freezer for 40–45 min. The female thrips were then removed from the freezer and allowed to defrost for at least 10 min. An individual dead female (virgin or mated) was placed in a new wax arena, as described above. A live virgin male (7–10 days old) was introduced individually to the dead virgin or dead mated female. Different males were used for every observation. The behaviours were recorded. Each treatment was replicated 14–16 times.

### Data analysis

Non-parametric tests were used throughout because the data were not normally distributed. The frequencies of behaviours were compared between treatments with Fisher’s exact test with the frequencies of pairs that did or did not exhibit the behaviour as one factor and the age or mating status as the other factor. Durations of behaviours were compared between treatments with a Mann–Whitney test with continuity correction or a Kruskal–Wallis test. Associations between variables were tested with a Spearman’s rank correlation test. Data were summarised with medians and ranges because they were non-parametric and highly skewed. All data were analysed with R 4.0.3^32^.

## Results

### Observations of mating behaviour

In laboratory observations, a male typically approached the female from the front or the side (Fig. 1a) and antennated her briefly. Some males walked away and avoided the female. Others attempted to climb on her back and were met with resistance, which took the form of the female running away, flipping the abdomen by as much as 90° and extreme twisting of the body. Males either walked away or they managed to hold on, bend the abdomen underneath the female abdomen and start copulating. For all pairs, the median duration of the first bout of resistance was 3 s (range 0–87 s, *n* = 80) and the median number of bouts was 1 (range 0–40 bouts, *n* = 80) with a median total duration of resistance of 5 s (range 0–225 s, *n* = 80). Female resistance occurred in all pairs in which the male started to climb on the female. A few seconds after the pair had started copulating, the female stopped resisting and the male antennated the female (median 3 s, range 0–7 s, *n* = 38) and stroked the dorsal part of the female abdomen and thorax with a midleg (median 3 s, range 0–9 s, *n* = 35) (Fig. 1b). After a short period from the start of copulation while the male was on top of the female (median 5 s, range 1–12 s, *n* = 42), the female moved away so that the pair formed a V-shape (Fig. 1c) and then both usually stayed still in this position for most of the copulation period. Sometimes the male continued stroking movements with its mid-legs, even though the female body was not within reach. The median duration of copulation was 154 s (range 96–643 s, *n* = 40). Copulation ended with the male walking away, which pulled on the female abdomen until the abdomens separated (Fig. 1d). An example of the whole process is shown in Supplementary Video S1.

Female resistance usually involved very fast movements by both the male and female. The flipping (Fig. 2a) and twisting sometimes caused males to end up underneath the female (Fig. 2b) or on top of the female holding the female upside down against the floor of the arena (Fig. 2c,d). During these struggles, males sometimes antennated the head of the female. After violent struggles, a few virgin males staggered in a way that suggested damage to the legs.

Field observations in a cowpea crop revealed male aggregations on leaves of cowpea in the morning and afternoon, which typically consisted of about 10 to 50 males. Aggregations formed on < 1% of leaves. Aggregations were also seen within the crop on the leaf of a tropical spiderwort *Commelina benghalensis* L. (family Commelinaceae), which is a common agricultural weed. Yellow and blue plastic containers placed within the crop had aggregations of as many as 200 males. No male-male fighting was observed.

The behaviour of pairs in the field showed the same features as in the laboratory, with some males making contact with females and leaving without attempting to mate, females resisting males, and copulating pairs spending a long time in the V-position. The median duration of copulation in the field was 145 s (range 137–167 s, *n* = 14) and was not significantly different from the durations in the laboratory with insectary-reared specimens (Mann–Whitney test, W = 288, *P* = 0.58).

### Do males choose mates by female age?

#### Experiment 1

We tested whether males can discriminate between females of two different ages (1–2 d or 7–10 d). Younger virgin males (1–2 days old) showed no significant difference in their behaviour towards the two ages of virgin female (Fisher’s exact test, *P* = 0.54), but there was a significant difference for the older virgin males (7–10 days old) (Fisher’s exact test, *P* = 0.005) (Table 1). Older males climbed on younger females significantly more than on older females (Fisher’s exact test, *P* = 0.04), which shows that males detected the difference between the females before climbing on. There was also no significant difference in the duration of the first bout of female resistance for the two ages of virgin female paired with younger males (Fisher’s exact test, *P* = 0.44), but there was a significant difference for the two ages of virgin female paired with older males (Fisher’s exact test, *P* < 0.001) (Table 1). In contrast, there was no significant difference between young and old females in the total duration of female resistance (Fisher’s exact test for younger males, *P* = 0.44; Fisher’s exact test for older males, *P* = 0.25) or copulation duration (Fisher’s exact test for younger males, *P* = 0.69; Fisher’s exact test for older males, *P* = 0.38) (Table 1).

**Table 1:** The effects of male and female age on mating behaviour.

Behaviour	Males (1–2 days)			Males (7–10 days)		
	Females (1–2 days) (*n* = 19)	Females (7–10 days) (*n* = 21)	*P*	Females (1–2 days) (*n* = 21)	Females (7–10 days) (*n* = 20)	*P*
Contacted	100	100	1.00	100	95	0.49
Climbed	84	90	0.65	95	70	0.04
Bent abdomen	79	76	1.00	95	58*	0.007
Copulated	58	48	0.54	76	30	0.005
Median duration of first female resistance (s)	5 (0–24)	3 (0–12)	0.44	6 (0–87)	1 (0–4)	< 0.001
Median duration of total female resistance (s)	7 (0–167)	4 (0–51)	0.44	7 (0–225)	4 (0–183)	0.25
Median copulation duration (s)	150 (96–237)	139 (117–201)	0.69	157 (117–643)	176 (139–384)	0.38

The percentage of male–female pairs that exhibited each behaviour in the sequence of mating behaviours, the median duration of the first bout of female resistance, the median duration of total female resistance and the median copulation duration for live virgin males (1–2 days or 7–10 days old) with live virgin females (1–2 days or 7–10 days old). *P*-values for behaviours were calculated using Fisher’s exact test for a 2 × 2 contingency table with the frequencies of pairs that did or did not exhibit the behaviour against whether the age of the female was 1–2 days or 7–10 days. Medians are given with ranges.

*P*-values for durations were calculated with Mann–Whitney tests.

*The observation of the behaviour was obscured briefly for one pair, so it was omitted from the analysis of that behaviour.

**Figure 3:**
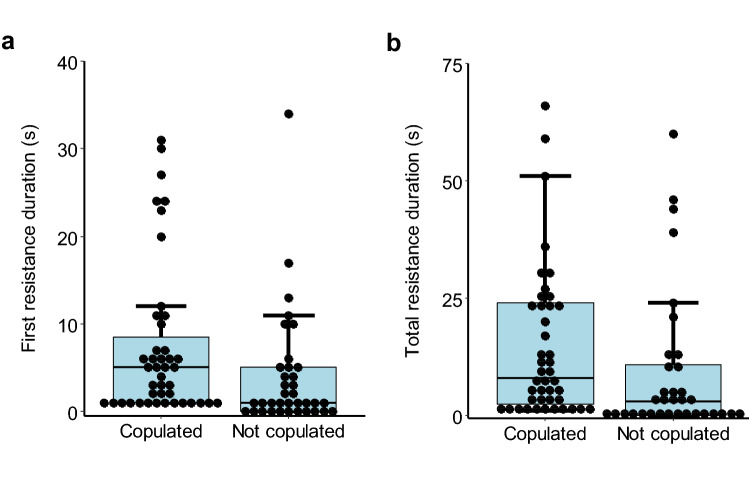
Box and whisker plots with superimposed dot plots of the durations of female resistance for pairs of *Megalurothrips sjostedti*, according to whether the pair copulated (*n* = 43) or did not copulate (*n* = 37). (**a**) Duration of the first bout of resistance (Mann–Whitney test, *W* = 526, *P* = 0.008). One outlier data point (87 s) for a pair that did not copulate is omitted in order to show the spread of the other points in more detail. (**b**) Duration of the total resistance (Mann–Whitney test, *W* = 587, *P* = 0.044). Three outlier data points (167 s, 183 s and 225 s) for pairs that did not copulate are omitted in order to show the spread of the other points in more detail.

If female resistance functions to deter males from copulating, the resistance should be longer for pairs that did not copulate. However, across all age combinations, there was significantly longer female resistance for pairs that copulated than for pairs that did not, whether female resistance was measured as the duration of the first bout of resistance (Mann–Whitney test, W = 526, *P* = 0.008) (Fig. 3a) or the total resistance (Mann–Whitney test, W = 587, *P* = 0.04) (Fig. 3b). However, lack of resistance could just be due to lack of persistence by males that did not attempt to mate. After omitting from the data all pairs for which there was no female resistance (11 pairs that did not attempt to copulate), there was no significant difference in female resistance between pairs that copulated and pairs that did not, for either the first bout of resistance (Mann–Whitney test, W = 526, *P* = 0.68) or the total resistance (Mann–Whitney test, W = 587, *P* = 0.73).

For pairs that copulated, there was no evidence that the amount of female resistance affected the duration of copulation. There was no significant correlation between the amount of female resistance and the subsequent copulation duration across all pairs that copulated (*n* = 40), whether measured as the duration of the first bout of resistance (Spearman rank correlation, ρ =  − 0.23, *P* = 0.15) or the total resistance (Spearman rank correlation, ρ =  − 0.04, *P* = 0.79).

#### Experiment 2

To test whether older males can discriminate between females of different ages (1–2 d or 7–10 d) without any behavioural cues from the females, such as differences in female resistance, we tested the male response to dead females. Virgin males differed in their behaviour towards the dead younger females and dead older females (Table 2). The difference was significant at the abdomen bending stage (Fisher’s exact test, *P* = 0.01), which shows the males could detect the difference before bending the abdomen. Most males walked away from the dead 7–10 days old females after contact or after climbing.

**Table 2:** The effects of female age on male mating behaviour.

Behaviour	Live virgin males (7–10 days)		
	Dead virgin females (1–2 days) (*n* = 18)	Dead virgin females (7–10 days) (*n* = 18)	*P*
Contacted	100	89	0.49
Climbed	89	56	0.06
Bent abdomen	89	44	0.01
Copulated	72	22	0.007

The percentage of male–female pairs that exhibited each behaviour in the sequence of mating behaviours for live virgin males with dead virgin females. The males were 7–10 days old and the females were either 1–2 days old or 7–10 days old.

*P*-values were calculated using Fisher’s exact test for a 2 × 2 contingency table with the frequencies of pairs that did or did not exhibit the behaviour against whether females were 1–2 days old or 7–10 days old.

### Do males choose mates by female mating status?

#### Experiment 3

To test whether older virgin males would avoid mating with previously mated females and whether mated females would mate again, we paired an initially virgin female with a succession of three different virgin males. There was no significant difference in the proportion of pairs that mated at each of the three introductions (Table 3). At each of the three introductions, most of the males (80–92%) mated with the females. Eight out of the original 12 females (67%) mated with a male at all three introductions. There was no significant difference in the copulation duration between the three introductions, so there was no sign that the first copulation by a female differed from subsequent copulations.

**Table 3:** The effects of female mating status on mating behaviour.

Behaviour	Successive introductions of males to females			
	1st (*n* = 12)	2nd (*n* = 11)	3rd (*n* = 10)	*P*
Contacted	100	100	100	1.0
Climbed	100	100	100	1.0
Bent abdomen	100	100	90	0.30
Copulated	92	91	80	0.66
Median copulation duration (s)	143 (115–552)	180 (120–279)	188 (131–270)	0.20

The percentage of male–female pairs that exhibited each behaviour in the sequence of mating behaviours and the median (range) copulation duration for virgin females introduced to three different virgin males in succession. The females were 1–2 days old for the 1st and 2nd introductions in immediate succession and 2–3 days old for the 3rd introduction after 24 h. The males were 7–13 days old. Only females that copulated during an introduction were tested in the next introduction.

*P*-values for behaviours were calculated using Fisher’s exact test for a 2 × 3 contingency table with the frequencies of pairs that did or did not exhibit the behaviour against the three introductions. The *P*-value for copulation duration was calculated with a Kruskal–Wallis test.

#### Experiment 4

To test whether older males can discriminate between virgin and mated females without any behavioural cues from the females, such as stronger resistance from mated females, we tested the male response to dead females. There was no significant difference between the proportion of virgin males that copulated with dead females that were virgin and the proportion that copulated with dead females that had mated once (Table 4). Most males copulated with the females, whether virgin or mated.

**Table 4:** The effects of female mating status on male mating behaviour.

Behaviour	Live virgin males (7–10 dsya)		
	Dead virgin females (1–2 days) (*n* = 14)	Dead mated females (1–2 days) (*n* = 15)	*P*
Contacted	100	100	1.00
Climbed	100	100	1.00
Bent abdomen	100	100	1.00
Copulated	79	53	0.25

The percentage of male–female pairs that exhibited each behaviour in the sequence of mating behaviours for live virgin males with either dead virgin females or dead mated females. The males were 7–10 days old and the females were 1–2 days old. *P*-values were calculated using Fisher’s exact test for a 2 × 2 contingency table with the frequencies of pairs that did or did not exhibit the behaviour against whether females were virgin or mated.

## Discussion

The most striking difference in mating behaviour between *M. sjostedti* and other thrips species was the resistance by virgin females. Abdominal flipping as a resistance mechanism has been described in several thrips species, but only by females that had mated previously or where there was an unusual size difference between male and female^6,16,28,33,34^ and not as the usual behaviour of virgin females. Resistance by virgin females is rare, but is also found in seaweed flies (Diptera: Coelopidae)^29^. Eberhard^35^ described two functions of female resistance, which are difficult to distinguish. From the female point of view, these are (1) resistance to avoid males indiscriminately (male coercion) and (2) female screening of suitable males (male persuasion). The former seems unlikely in *M. sjostedti* because females would not seek out male aggregations if they were avoiding males. Screening of males implies a selective co-operation by the female and there is some evidence for this because the female always stopped all resistance within seconds of starting to copulate. In contrast, *E. americanus* can continue resisting and reject males for 10–20 s after copulation starts^33^. The considerable variation in the duration of female resistance between pairs (Table 1, Fig. 3) could indicate screening, but could also just reflect variation in male persistence. There was no evidence that there was more or less female resistance for pairs that copulated than for pairs that did not, after omitting pairs in which the male appeared to make no attempt to mate. Nor was there any correlation between female resistance and the subsequent duration of copulation, which could have indicated a form of cryptic male choice^36^ or cryptic female choice^37^ by influencing copulation duration. Thus, there is no compelling evidence for or against the hypothesis that female resistance screens males as a form of female choice.

A third possible function of female resistance is that it prevents the male from applying an anti-aphrodisiac pheromone (AAP) when on the back of the female. Such pheromones are known or strongly indicated in two other thrips species in the same family^20,38^. The method of applying AAP in thrips is not known, but it appears to be produced and thus applied from the male head-thorax region^38^ and thus not by genital contact. After female resistance, *M. sjostedti* males spend most time during copulation in the V-position. This is the predominant male position also in the common blossom thrips *F. schultzei* (Trybom)^7^, but males spend most time on the back of the female in *F. occidentalis*^34^ and *E. americanus*^39^. The result of being mostly in the V-position was that stroking the female, which could be the method of applying an AAP, lasted only 3 s (2% of the copulation time) in *M. sjostedti*, whereas in *F. occidentalis* it was 130 s (56% of the copulation time)^28^. The AAP-avoidance hypothesis requires there to be a sexual conflict in which multiple mating is advantageous to females^40,41^ and repeat mating by a male with the same female is disadvantageous to males. There was no evidence that females avoided repeat copulations or that such copulations ended prematurely, since females mated multiple times and the copulation duration for first and repeat copulations were similar (Table 3). This contrasts with *F. occidentalis*, in which repeat copulations were consistently much shorter, suggesting that they were ineffective^28,34^. Much relevant information, such as whether repeat matings are effective or whether mated females return to male aggregations or are attracted to the aggregation pheromone, is not known with certainty for any thrips species, although response to aggregation pheromone by mated females seems likely in *F. occidentalis*^42^. Not enough is known about the mating system in *M. sjostedti* to draw conclusions about this hypothesis.

In field observations, male aggregations were not confined to leaves of the host-plant but were found also on leaves of a non-host species and particularly on brightly coloured plastic surfaces. *Megalurothrips sjostedti* is already known to be attracted to coloured traps^43^. It is likely that these responses are similar for aggregating and non-aggregating individuals, as has been shown for *F. occidentalis*^44^. Leaves may be the usual aggregation site only because a more visually apparent surface is not available in the crop. The host flowers are small and not very obvious. This is similar to the behaviour of the closely related *P. kellyanus*, in which males aggregate on citrus leaves but also on mature lemon fruit when present^16^. There is still much that is unknown about the aggregations in *M. sjostedti*, such as whether the same sites are used on successive days and whether female arrival rate increases with aggregation size.

Older males copulated or attempted to copulate less often with older females (Tables 1, 2). This is similar to the greater discrimination of female mating status by experienced males compared with virgin males in *F. occidentalis*^20^. A similar decline in male mating attempts with female age for mated females was found in *E. americanus*^33^, but not in *T. tabaci*^45^. Male *M. sjostedti* discriminated female age even when females were dead, so the effect is clearly male choice.

An obvious reason for avoiding mating with older females is that their potential future fecundity is reduced because they have less long to live. However, males in aggregations and in our experiments were choosing whether or not to mate with females in succession and not choosing between young and old females present at the same time. This suggests that the benefits of not mating outweigh the cost of lost mating opportunities^22,24^, perhaps because of limited resources for mating. Males discriminated after initial contact and before climbing on the female, which suggests that the initial antennation was detecting a difference. Males could be using cuticular hydrocarbons as a cue to female age. Cuticular hydrocarbons change qualitatively and quantitatively with age in many insect groups^46–48^. Older males were more discriminating than younger males. Since the males were all virgins, this could not be explained by learning or increased mating experience. Older males have less long to live on average and might therefore be expected to become less choosy and less likely to miss a mating opportunity rather than more. The effect may be the result of age-dependent increases in olfactory sensitivity^49^. The degree of male choosiness might also be influenced by the number of females arriving at an aggregation.

There was no significant effect of female mating status on male mating frequency (Table 4). Any effect, if present, must be rather weak. A similar experiment with dead females in *F. occidentalis* showed a very clear reduction in the proportion of males mating with females that had mated, and was considered likely to be the effect of an AAP^20^. The lack of a strong response in *M. sjostedti* may reflect the lack of opportunity to apply AAP, as discussed above, and the response instead to female age may be a proxy for detecting whether females are likely to have mated.

In conclusion, we found that older males choose females by age, whereas any choice by female mating status was weak or absent. Evidence for female choice remains elusive. Study of the mating behaviour of *M. sjostedti* has revealed broad similarities with other thrips species that form aggregations, but also some distinct and novel differences.

## Supplementary Information

Supplementary Legend.

Supplementary Video S1.

## Data Availability

The data that support the findings of this study are available from the corresponding author upon reasonable request.
