# Supplementary material for: Mating behaviour, mate choice and female resistance in the bean flower thrips (Megalurothrips sjostedti)
Source: Sci Rep. 2021 Jul 15;11:14504. doi: 10.1038/s41598-021-93891-5 (PMC8282879; doi:10.1038/s41598-021-93891-5)
Supplement: Supplementary file 1 — Supplementary Legend. [file 41598_2021_93891_MOESM1_ESM.docx]

**Supplementary Information.** Supplementary Video S1. Mating behaviour of male and female bean flower thrips (*Megalurothrips sjostedti*), including initial female resistance.
